# Blocking the Function of Inflammatory Cytokines and Mediators by Using IL-10 and TGF-β: A Potential Biological Immunotherapy for Intervertebral Disc Degeneration in a Beagle Model

**DOI:** 10.3390/ijms151017270

**Published:** 2014-09-26

**Authors:** Wei Li, Tianyi Liu, Liangliang Wu, Chun Chen, Zhiwei Jia, Xuedong Bai, Dike Ruan

**Affiliations:** 1 Department of Orthopedics, Navy General Hospital, Beijing 100048, China; E-Mails: weilichn@126.com (W.L.); chenchunkk@163.com (C.C.); jiazhiweivip@163.com (Z.J.); baixuedongngh@163.com (X.B.); 2 Department of Centre Laboratory, General Hospital of People’s Liberation Army, Beijing 100853, China; E-Mails: tianyiliu08@163.com (T.L.); wuliang080228@163.com (L.W.)

**Keywords:** intervertebral disc degeneration, inflammation, interleukin-10, transforming growth factor-β, cytokines

## Abstract

The debilitating effects of lower back pain are a major health issue worldwide. A variety of factors contribute to this, and oftentimes intervertebral disk degeneration (IDD) is an underlying cause of this disorder. Inflammation contributes to IDD, and inflammatory cytokines such as tumor necrosis factor (TNF)-α and interleukin (IL)-1β, play key roles in the pathology of IDD. Therefore, the development of treatments that inhibit the expression and/or effects of TNF-α and IL-1β in IDD patients should be a promising therapeutic approach to consider. This study characterized the potential to suppress inflammatory cytokine production in degenerative intervertebral disc (NP) cells by treatment with IL-10 and TGF-β in a canine model of IDD. IDD was induced surgically in six male beagles, and degenerative NP cells were isolated and cultured for *in vitro* studies on cytokine production. Cultured degenerative NP cells were divided into four experimental treatment groups: untreated control, IL-10-treated, TGF-β-treated, and IL-10- plus TGF-β-treated cells. Cultured normal NP cells served as a control group. TNF-α expression was evaluated by fluorescence activated cell sorting (FACS) analysis and enzyme-linked immunosorbent assay (ELISA); moreover, ELISA and real-time PCR were also performed to evaluate the effect of IL-10 and TGF-β on NP cell cytokine expression *in vitro*. Our results demonstrated that IL-10 and TGF-β treatment suppressed the expression of IL-1β and TNF-α and inhibited the development of inflammatory responses. These data suggest that IL-10 and TGF-β should be evaluated as therapeutic approaches for the treatment of lower back pain mediated by IDD.

## 1. Introduction

Debilitating lower back pain (LBP) is a significant medical problem for over half of the adult population worldwide [1]. A key factor that contributes to LBP is intervertebral disc degeneration (IDD), which can result in debilitating pain and reduce the quality of life for LBP patients as well as inflicting significant financial burdens due to lost productivity and increased health care costs [2,3]. Effective clinical management of LBP caused by IDD is challenging, because the exact mechanism of IDD is still unclear, and current therapies for LBP caused by IDD only address symptomatic relief for the patient and not the underlying cause of the disease [4]. A better understanding of the mechanisms that cause IDD are needed to enable the development of effective disease-modifying therapies that block the biochemical and pathophysiologic processes mediating this degeneration [5,6]. Biological therapeutic approaches appear to be promising strategies to consider since proinflammatory cytokines play a role in the development of IDD [7].

IDD can be triggered and exacerbated by several factors, including mechanical stress, trauma, genetic predisposition, and local inflammation [8]. Intradiscal cells, particularly nucleus pulposus (NP) cells, play an important role in maintaining the biomechanical functions of the spine and intervertebral disc height. Mechanical stress can increase the release of various cytokines within the intravertebral disk (IVD) region, causing pain, inflammation and tissue damage [9]. Compared with acute disc injury, IDD is characterized by a gradual decrease in tissue hydration, especially in the NP, suggesting a transition to a less fluid-like elastic material in the nucleus after neovascularization and nerve ingrowth occurs in the typically avascular disk. The water content decreases from 90% wet weight of the nucleus tissue to less than 70% [8]. More importantly, inflammation contributes to IDD [10] and the inflammatory cytokines produced are associated with progression of IDD [11,12,13]. A successful therapeutic approach should promote anabolism and cell proliferation in addition to controlling inflammation and the resulting tissue damage.

Tumor necrosis factor (TNF)-α significantly reduces anabolism in IVD and induces cell senescence [14]; it facilitates catabolic processes leading to extracellular matrix breakdown in IVDs by decreasing the expression of genes encoding proteoglycans and type II collagen, which are major structural components of IVDs. Matrix metalloproteinases (MMPs) are key mediators of extracellular matrix deterioration, and have been implicated in IDD [15], and studies have shown that TNF-α increases the secretion of MMPs in humans [16].

Interleukin (IL)-1β plays a crucial role in IDD by inducing proteoglycan breakdown and inhibiting matrix biosynthesis by IVD cells [17]. IL-1β is produced in response to infection, injury, and antigenic changes and has been shown to induce apoptosis of IVD cells [18]. Studies using cell cultures from human intervertebral discs have shown that IL-1β interacts with TNF-α [19]. IL-1β also increases catabolic enzyme activity [20], and other studies in human systems have shown that IL-1β inhibitors can affect signaling pathways in models of IDD [21].

IL-10 inhibits inflammatory cytokine synthesis and is has been reported that *in vitro* TNF-α secretion in cells is suppressed more than 90% after IL-10 treatment [22]. The importance of IL-10 in inhibiting inflammation was demonstrated by the phenotype of IL-10–null mice, which develop inflammatory lesions in the intestinal tract [23]. These data show that IL-10 plays an important role in modulating the expression of immune effector molecules [24].

Numerous studies have shown the protective effect of transforming growth factor (TGF)-β in IDD. Most of these have focused on the ability of TGF-β to increase proteoglycan production, type II collagen levels, and IVD cell proliferation, as well as its ability to reduce matrix degradation and regulate disc cell metabolism [25,26,27]. An earlier study from our laboratory reported that TGF-β induces proliferation of IVD cells when human NP cells were evaluated [28] and TGF-β gene therapy with rabbit NP cells found increases in proteoglycan production [29]. TGF-β also has key anti-inflammatory properties, such as suppressing the ability of monocytes/macrophages to release inflammatory cytokines [30]. Additionally, the transfer of plasmid DNA encoding TGF-β was shown to suppress inflammatory lesions in a rat model of arthritis [31]. TGF-β also up-regulates IL-10 synthesis by mouse macrophages and rat hepatic stellate cells [32,33].

Although IL-10 and TGF-β can inhibit synthesis of inflammatory cytokines, few studies have evaluated the therapeutic potential of IL-10 and TGF-β as anti-inflammatory mediators in IDD. We evaluated the potential of IL-10 and TGF-β to inhibit the release of TNF-α and IL-1β from degenerative NP cells to determine if combining these two biologics might be a promising approach for the treatment of IDD.

## 2. Results

### 2.1. Intracellular Inflammatory Cytokine Analysis with Flow Cytometry

**Figure 1:**
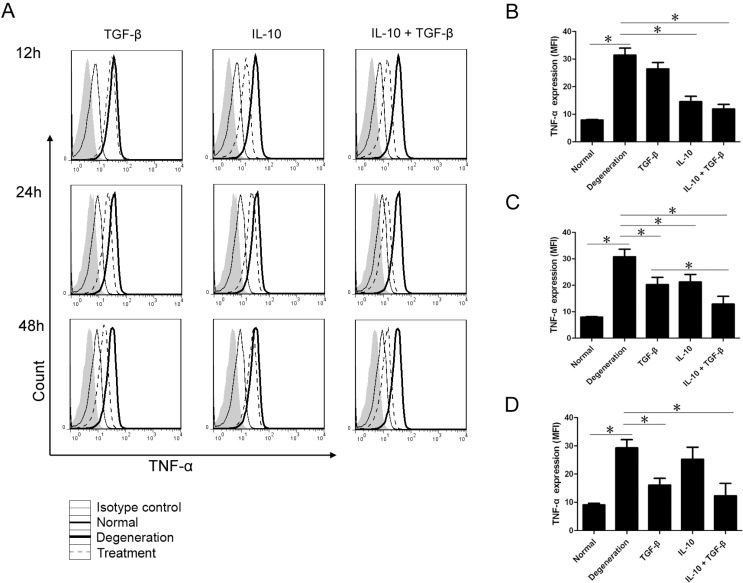
The cell counts and the mean fluorescence intensity (MFI) of intracellular inflammatory cytokines. Nucleus pulposus (NP) cells were divided into five treatment groups: untreated normal NP cells, untreated degenerative NP cells, interleukin (IL)-10 (20 ng/mL) treated, transforming growth factor (TGF)-β (20 ng/mL) treated, and IL-10 (20 ng/mL) + TGF-β (20 ng/mL) treated degenerative NP cells. Each group was treated for 12, 24, or 48 h in the culture medium before analysis. Cells expressing tumor necrosis factor (TNF)-α 12–48 h after treatment with cytokines are shown in (**A**); At 12 h, few cells were positive, and a lower level of TNF-α (MFI) was expressed in the untreated normal NP cells group (7.92 ± 0.32), IL-10 (14.57 ± 3.37), and IL-10 + TGF-β (11.9 ± 2.91) groups as compared to the untreated degenerative NP cells group (31.47 ± 4.38) (**B**); At 24 h, the MFI values in the normal NP cells group (7.94 ± 0.22) and all three treatment groups (IL-10: 21.23 ± 2.85; TGF-β: 20.27 ± 2.76; IL-10 + TGF-β: 12.9 ± 2.91) were significantly lower than in the untreated degenerative NP cells group (30.8 ± 2.86) (**C**); At 48 h, a lower level of TNF-α (MFI) was seen in the normal NP cells group (9.07 ± 0.54), TGF-β group (16.07 ± 2.46) and the IL-10 + TGF-β group (12.3 ± 4.42), as compared with the untreated degenerative NP cells group (29.3 ± 2.84) (**D**). Pairwise comparisons between each group were analyzed using the Bonferroni correction, and the mean ± SD was determined. *p* < 0.05 was considered statistically significant. Data are representative of six independent experiments from different animals. Data are the means ± SD of triplicate cultures. * *p* < 0.01.

NP cells were divided into five treatment groups: Normal NP cells; untreated degenerative NP cells; and IL-10-treated, TGF-β-treated, and IL-10 + TGF-β-treated degenerative NP cells. The cell count and the mean fluorescence intensity (MFI) of each group were determined by flow cytometry at different time points (Figure 1). Cells expressing TNF-α 12–48 h after treatment with cytokines are shown in Figure 1A. Twelve hours after treatment, compared with untreated degenerative NP cells, the number of cells positive for TNF-α in the IL-10 and IL-10 + TGF-β treatment groups decreased sharply (Figure 1A). The expression of TNF-α (MFI) in untreated normal NP cells as well as in IL-10 and IL-10 + TGF-β treated NP cells was significantly lower (*p* < 0.01) compared with the untreated degenerative NP cell group (Figure 1B). After 24 h, the MFI values for TNF-α in the normal NP cells group and all three treatment groups were significantly lower (*p* < 0.01) relative to untreated degenerative NP cells. Treatment with both IL-10 and TGF-β resulted in significantly lower TNF-α expression (*p* < 0.01) than treatment with TGF-β or IL-10 alone (Figure 1C), and TNF-α levels were similar to levels observed in the normal NP cells group. The number of cells positive for TNF-α in the TGF-β group and the IL-10 + TGF-β group reached their lowest levels 48 h after treatment (Figure 1A), and the MFI values in these two groups were significantly lower (*p* < 0.01) compared with untreated degenerative NP cells, resulting in TNF-α levels that were similar to levels for untreated normal NP cells (Figure 1D).

### 2.2. Quantitative Assay of Cytokines in the Supernatant

**Figure 2:**
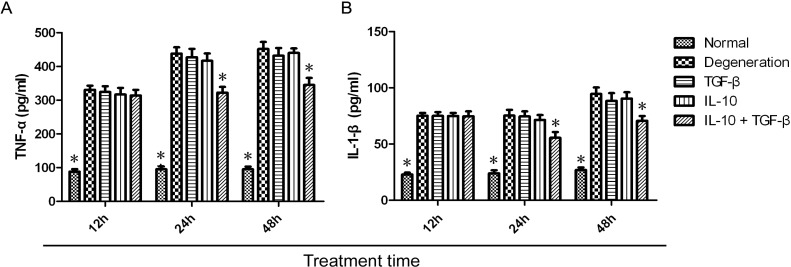
Quantitative expression of IL-1β and TNF-α in supernatants. NP cells were divided into five treatment groups: untreated normal NP cells, untreated degenerative NP cells, IL-10 (20 ng/mL) treated, TGF-β (20 ng/mL) treated, and IL-10 (20 ng/mL) + TGF-β (20 ng/mL) treated cells. Degenerative NP cells were treated for 12, 24, or 48 h in the culture medium before analysis. TNF-α (**A**) and IL-1β (**B**) levels in the IL-10 + TGF-β group (TNF-α: 322.12 ± 17.07, 24 h, and 345.19 ± 20.83, 48 h; IL-1β: 55.53 ± 5.1, 24 h, and 70.72 ± 4.24, 48 h) were significantly lower (*p* < 0.01) than the corresponding levels in the untreated degenerative NP cell group (TNF-α: 438.06 ± 18.9, 24 h, and 451.94 ± 20.91, 48 h; IL-1β: 75.29 ± 5.12, 24 h, and 94.51 ± 5.77, 48 h) at 24 and 48 h. TNF-α (**A**) and IL-1β (**B**) levels in untreated normal NP cells group (TNF-α: 88.2 ± 7.5, 12 h; 95.13 ± 8.5, 24 h; and 95.2 ± 7.98, 48 h; and IL-1β: 22.9 ± 1.65, 12 h; 23.88 ± 2.76, 24 h; and 26.94 ± 2.21, 48 h) were significantly lower (*p* < 0.01) than the corresponding levels in untreated degenerative NP cell group (TNF-α: 330.55 ± 12.73, 12 h; 438.06 ± 18.9, 24 h; and 451.94 ± 20.91, 48 h; and IL-1β: 75.17 ± 2.53, 12 h; 75.29 ± 5.12, 24 h; and 94.51 ± 5.77, 48 h) at all three time points. Pairwise comparisons between each group were carried out using the Bonferroni method, and the mean ± SD was determined. *p* < 0.05 was considered statistically significant. Data are representative of six independent experiments from different animals. Data are the means ± SD of triplicate cultures. * *p* < 0.01.

Using an enzyme-linked immunosorbent assay (ELISA), we measured IL-1β and TNF-α levels in the supernatant of normal NP cells in each treatment group at 12, 24, and 48 h. These levels were compared to the levels expressed by untreated degenerative NP cells. In the IL-10 + TGF-β group, TNF-α (Figure 2A) and IL-1β (Figure 2B) levels were significantly lower at 24 and 48 h, as compared with the untreated degenerative NP cells group. IL-1β levels were the lowest 24 h after treatment and slightly increased at 48 h. IL-1β and TNF-α levels in the IL-10 + TGF-β treatment group were significantly lower (*p* < 0.01) at 24 and 48 h, whereas the corresponding levels in the normal NP cells group were significantly lower (*p* < 0.01) at 12, 24 and 48 h.

### 2.3. Quantitative Analysis of mRNA Expression

**Figure 3:**
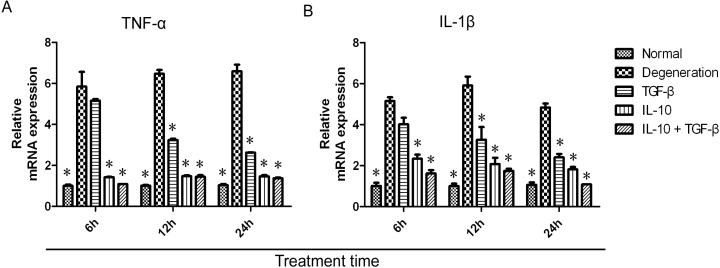
mRNA expression of TNF-α and IL-1β. NP cells were divided into five groups: untreated normal NP cells, untreated degenerative NP cells, IL-10 (20 ng/mL), TGF-β (20 ng/mL), and IL-10 (20 ng/mL) + TGF-β (20 ng/mL) degenerative NP cell treatment groups. Treatment was for 6, 12, or 24 h in the culture medium before analysis. Total RNA was extracted from cells and reverse transcribed to synthesize cDNA. mRNA expression for TNF-α (**A**) and IL-1β (**B**) was quantified by real-time PCR relative to the β-actin housekeeping gene. The data for mRNA expression are shown as 2-ΔΔ*C*_t_ values. Compared with the degenerative NP cells group (TNF-α: 5.85 ± 0.71, 6 h; 6.47 ± 0.19, 12 h; and 6.59 ± 0.33, 24 h; and IL-1β: 5.15 ± 0.19, 6 h; 5.91 ± 0.43, 12 h; and 4.83 ± 0.2, 24 h), the TNF-α and IL-1β mRNA levels in the normal NP cells group (TNF-α: 1 ± 0.06, 6 h; 1.01 ± 0.04, 12 h; and 1.03 ± 0.06, 24 h; and IL-1β: 1.01 ± 0.15, 6 h; 1 ± 0.13, 12 h; and 1.07 ± 0.12, 24 h), IL-10 group (TNF-α: 1.42 ± 0.02, 6 h; 1.47 ± 0.04, 12 h; and 1.46 ± 0.06, 24 h; and IL-1β: 2.34 ± 0.2, 6 h; 2.07 ± 0.31, 12 h; and 1.82 ± 0.12, 24 h) and the IL-10 + TGF-β group (TNF-α: 1.08 ± 0.02, 6 h; 1.44 ± 0.08, 12 h; and 1.36 ± 0.05, 24 h; and IL-1β: 1.62 ± 0.16, 6 h; 1.74 ± 0.11, 12 h; and 1.08 ± 0.1, 24 h) were significantly lower (*p* < 0.01) at 6, 12, and 24 h. Pairwise comparisons between each group were analyzed using the Bonferroni method and the mean ± SD was determined. *p* < 0.05 was considered statistically significant. Data are representative of six independent experiments from different animals. Data are the means ± SD of triplicate cultures. * *p* < 0.01.

mRNA transcript levels in cells from each treatment group and control groups were quantified by real-time PCR. The expression of TNF-α and IL-1β mRNA was suppressed by treatment with IL-10 and TGF-β (Figure 3A,B). After treatment with TGF-β, the expression of TNF-α and IL-1β mRNA was maximal at 6 h and then decreased gradually from the 6 to 24 h time points. Compared with untreated degenerative NP cells, TNF-α and IL-1β mRNA levels (2^−ΔΔ*C*t^ values) in untreated normal NP cells and the TGF-β treatment group were significantly lower *(p* < 0.01) at 12 and 24 h. In contrast, a rapid decrease in TNF-α and IL-1β mRNA levels in the IL-10 or IL-10 + TGF-β treatment groups was seen at 6 h and persisted up to the 24 h time point. Compared with untreated degenerative NP cells, TNF-α and IL-1β mRNA levels in untreated normal NP cells, IL-10, and IL-10 + TGF-β treatment groups were significantly lower (*p* < 0.01) at 6, 12, and 24 h.

## 3. Discussion

Debilitating lower back pain is a major medical issue in adults. IDD is a natural phenomenon of the aging spine that can be triggered by various stimuli, but ultimately have a proinflammatory aspect that causes tissue damage in patients. The intervertebral disc is an avascular tissue consisting of poorly characterized cells in an extracellular matrix. The annulus fibrosus (AF) consists of discrete concentrically organized layers of fibrous tissue or lamellae that surround the NP in a normal disc. The NP and AF constitute the primary load-bearing system of the spinal column in humans [8]. While NP cells regulate homeostasis of IVD tissues by maintaining a balance between anabolism and catabolism [34], AF-associated cells also play a key role in degenerative and repair processes. A study suggested that AF cells are involved in an inflammatory reaction and interactions between AF and neuron-like cells enhances the production of growth factors responsible for neovascularization and nerve ingrowth that supports the development of IDD [35].

Because of the role of proinflammatory cytokines in the development of IDD, biological therapy is considered a promising treatment strategy for this indication. TNF-α and IL-1β are the primary inflammatory mediators of IDD and its progression, playing a role in inhibition of anabolic processes required for IVD maintenance and repair. Additionally, these factors have been reported to induce apoptosis of native IVD cells [14,18]. Various other mediators of tissue damage are upregulated as a consequence of cytokine-mediated inflammation. These include matrix metalloproteinases, which are produced following IL-1β and TNF-α stimulation and contribute to breakdown of extracellular matrix components present in IVD structures. There is a robust set of data that proves the role TNF-α and IL-1β as key inflammatory factors leading to the development of IDD, and identifying molecules that can inhibit the production of these cytokines could be an effective mode of treatment for IDD. Previous studies showed that IL-10 and TGF-β inhibit macrophage production of the inflammatory cytokines TNF-α and IL-1β [36], and it was therefore of interest to determine if these effects were also observed in other cell types that can produce IL-1β and TNF-α.

In our study, TGF-β acted slowly, requiring 12 to 24 h to exert an inhibitory effect, which is consistent with suppression of the translation of cytokine mRNA. IL-10 acted at an early step in cytokine production and markedly suppressed TNF-α and IL-1β mRNA levels, having a more rapid effect than TGF-β, showing inhibition of cytokine expression as early as 6 h after treatment. Previous studies demonstrated that the beneficial effects of TGF-β are varied, including increased proteoglycan production, collagen type II levels, proliferation of IVD cells, and decreasing matrix degradation as well as regulating disc cell metabolism [25,26,27,28]. The experiments described here demonstrated that TGF-β and IL-10 have anti-inflammatory properties in IDD, suppressing IL-1β and TNF-α production, and that there is an interaction between TGF-β and IL-10 since the combination of the two has greater effect than either as a single agent. Cytokine mRNA expression and intercellular cytokine levels measured by fluorescence activated cell sorting (FACS) analysis demonstrated that the expression of inflammatory cytokines in degenerative IVDs can be blocked by using exogenous TGF-β and IL-10, which could have therapeutic benefit in this disorder.

We observed that either TGF-β or IL-10 alone suppressed the expression of inflammatory cytokines. Furthermore, their combined use produced a higher level of inhibition of TNF-α and IL-1β than either TGF-β or IL-10 alone. There was a cumulative effect on the levels of IL-1β and TNF-α in the supernatant over time in culture. We believe this was due to evaporation of the culture medium over time, while the degenerative NP cells were continuously secreting inflammatory cytokines. IL-1β levels in the supernatant were significantly lower at 24 and 48 h, and the greatest decrease was seen 24 h after treatment in the IL-10 + TGF-β group. Although secretion of TNF-α and IL-1β was slightly decreased by the presence of TGF-β or IL-10 alone, no statistically significant difference was seen between either of these two cytokine treatment groups and the degenerative NP cells group. However, compared with the degenerative NP cells, the expression of TNF-α and IL-1β in normal NP cells was significantly lower. Thus, regarding IL-1β and TNF-α in the supernatant, TGF-β may share some anti-inflammatory properties with IL-10. This concept is in agreement with a previous report on their effects in macrophages [37].

Results from real-time PCR showed that TNF-α and IL-1β mRNA levels decreased rapidly after 6 h of treatment with IL-10 and TGF-β, and the expression of mRNA was maintained at a minimal level from the 6 to 24 h time points. In contrast to TGF-β treatment alone, we observed a rapid decrease in TNF-α and IL-1β mRNA levels at all time points after treatment with IL-10 alone. This observation indicates that IL-10 acted at an early step in cytokine production. According to our flow cytometry analysis of intracellular inflammatory cytokines, the number of cells that were positive for TNF-α in cultured normal NP cells was not significantly different compared with the isotype control, showing that normal NP cells produce low levels of TNF-α. The MFI in untreated normal NP cells and IL-10 + TGF-β treated degenerative NP cells was significantly lower compared with untreated degenerative NP cells. The major findings of the this analysis are that after treatment with IL-10 and TGF-β, the expression of intracellular TNF-α and IL-1β was suppressed, while the expression of inflammatory cytokines in untreated normal NP cells was significantly lower than that in untreated degenerative NP cells.

This study has some limitations: in this canine model of IDD, the lower lumbar discs were injured (Lumbar (L) 2–L5), while the upper (L1–L2) lumbar disc was left un-injured and served as a control. While no direct acute mechanical stress was performed on this disc, it is considered a “relatively” normal disc versus a completely normal disc from an animal that has had no vertebral injury because degenerative discs can impact adjacent healthy disks and modify their baseline production of cytokines.

## 4. Experimental Section

### 4.1. Animals

Six healthy 1-year-old male beagle dogs (10–10.5 kg) were used. Animals were purchased from the Experimental Animal Center of PLA Navy General Hospital (Beijing, China). All animals were housed in a pathogen-free animal facility and maintained in accordance with the Committee on the Ethics of Animal Experiments and national guidelines on the care and use of laboratory animals. Digital radiography (4 mA, 68 KV) was performed to exclude animals with vertebral abnormalities (osteophyte formation, endplate calcification, Schmorl’s node, or a variation in the number of lumbar vertebral bodies) in the spine. T2-weighted magnetic resonance images (1.5 T) of lumbar discs in the sagittal plane with a spine coil were obtained (spin echo sequence with time to repetition of 2500 ms and time to echo of 85 ms) and animals with signs of lumbar disc degeneration were excluded from the study. All surgical procedures and postoperative care were approved by the Institutional Animal Ethics Committee of PLA Navy General Hospital (2013077, 9 January 2013). The protocol was approved by the Committee on the Ethics of Animal Experiments at the PLA Navy General Hospital (2013-B-056).

### 4.2. Animal Model of Intervertebral Disk Degeneration (IDD)

**Figure 4:**
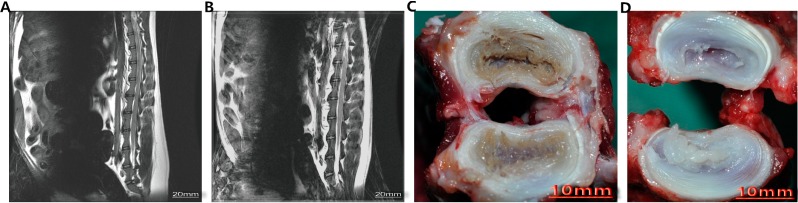
The T2-weighted magnetic resonance imaging (MRI) and morphology of the beagle model of Intervertebral Disk Degeneration (IDD). (**A**) The MRI of the lumbar disc after disc puncture; (**B**) The MRI of the lumbar disc without disc puncture; (**C**) The morphology of the lumbar disc after disc puncture; (**D**) The morphology of the normal lumbar disc.

Beagles were anesthetized with methoxyflurane (induction: 3% in 100% O_2_; maintenance, 0.5%–1%). Onset of anesthesia was checked by loss of the palpable reflex and pinprick sensation over the corresponding operative areas. Surgery commenced within 10 min of the onset of anesthesia. The beagle model of IDD was performed as described by Elliot *et al.* [38]. A surgical incision was made in each animal posterolateral from L2 to L5, followed by an annular puncture with a 16-gauge needle on the L2–L3, L3–L4, and L4–L5 discs. To grade the severity of IDD, T2-weighted magnetic resonance imaging studies were conducted on all animals after disc puncture and revealed substantial disc degeneration 6 weeks later. Similar changes were seen in all study animals at this time point (Figure 4).

### 4.3. Isolation and Culture of Nucleus Pulposus (NP) Cells

Once substantial disc degeneration was confirmed upon magnetic resonance imaging analyses, the dogs were sacrificed with an overdose of ketamine hydrochloride and xylazine hydrochloride injection. The L1–L2, L2–L3, L3–L4, and L4–L5 intervertebral discs were incised under aseptic conditions and NP tissues were obtained. L2–L3, L3–L4, and L4–L5 NP tissues were evenly mixed as integral degenerative NP tissues for further cell culture. The L1–L2 discs were used to obtain normal cells (no annular puncture). NP cells were then isolated as described by Chelberg *et al.* [39]. Briefly, NP tissues were diced into 2-mm^3^ pieces and digested in 0.2% pronase (Sigma-Aldrich, St. Louis, MO, USA) for 60 min at 37 °C and then digested overnight in 0.02% type II collagenase (Sigma-Aldrich). Cells were cultured in 75-cm^2^ cell culture flasks in a culture medium composed of equal parts of Dulbecco’s modified Eagle medium and Ham’s F-12 medium (DMEM/F12; HyClone, Logan, UT, USA), 10% fetal calf serum, and 1% penicillin/streptomycin for 7–10 days until confluent. The culture medium was changed every 3 days. All NP cells were used within the first three passages. A total of 2 × 10^7^ NP cells were obtained from the L1–L2 normal disc, and a total of 6 × 10^7^ NP cells were obtained from the L2–L5 combined degenerative discs.

### 4.4. Cytokine Treatments and Groups

To investigate the effect of cytokines, cultured degenerative NP cells were divided into four treatment groups (*n* = 6 per treatment group, one for each animal: untreated degenerative NP cell group; degenerative NP cells treated with IL-10 (IL-10 group) or TGF-β (TGF-β group) or both (IL-10 + TGF-β group) for 6, 12, 24, or 48 h at a concentration of 20 ng/mL for each cytokine. Untreated normal NP cells from the L1–L2 disc (*n* = 6, one for each animal) served as the negative control group.

### 4.5. Assay of Inflammatory Cytokine-Producing Cells

To analyze the expression of intracellular inflammatory cytokines at the various time points after treatment with cytokine(s), cells from each group were counted and cell suspension was added to Eppendorf tubes (2 × 10^5^ cells/tube and centrifuged at 3000 rpm for 3 min. Fluorescein isothiocyanate-labeled anti-TNF-α monoclonal antibodies (BD Pharmingen, Franklin Lakes, NJ, USA) were added to the tubes and the contents mixed thoroughly. Each tube was incubated in the dark at 4 °C for 30 min. Cells were washed with phosphate-buffered saline (PBS; Taigemei Biotechnology Co., Beijing, China), and analyzed by flow cytometry (FC500 MPL, BeckmanCoulter, Brea, CA, USA). Up to 10,000 NP cells were counted per tube. Data analysis was performed using FlowJo software (TreeStar Inc., Ashland, OR, USA). The MFI was calculated to detect NP cells expressing TNF-α.

### 4.6. Quantitative Assay of Cytokines in the Supernatant

After treatment with cytokines for 12, 24, and 48 h, IL-1β and TNF-α in the culture supernatants of each group were measured with enzyme-linked immunosorbent assay kits (ELISA Kit for IL-1β and TNF-α, BD Pharmingen) for quantification. The detection sensitivity limit was at least 5 pg/mL for IL-1β and TNF-α.

### 4.7. Real-Time PCR Analysis

To analyze the mRNA expression levels of IL-1β and TNF-α, total RNA was extracted from NP cells from each treatment group (5 × 10^5^ cells per plate; after treatment with cytokines for 6, 12, and 24 h as described above) using an RNeasy plus mini kit (Qiagen, Valencia, CA, USA). Genomic DNA was digested using an RNase-Free DNase kit (Qiagen). First-strand cDNA was synthesized using oligo-dT primers (Invitrogen, Carlsbad, CA, USA) and the Omniscript RT kit (Qiagen). The transcripts were quantified with real-time PCR using an ABI PRISM 7500 Sequence Detector (Applied Biosystems, Foster City, CA, USA) with Applied Biosystems predesigned TaqMan Gene Expression Assays (dog TNF-α, Cf02628237_mL; dog IL-1β, Cf02671952_mL) and reagents according to the manufacturer’s instructions. For relative quantification, gene expression in the samples was normalized to β-actin mRNA expression (dog β-actin, Cf03034055_uL) using the 2^−∆∆*C*t^ method.

### 4.8. Statistical Analysis

The data were analyzed using Stata 12.0 statistical software for single-factor ANOVA (Purchased from StataCorp, Lakeway, TX, USA). Pairwise comparisons between each group were analyzed with the Bonferroni method, and the mean ± SD was determined. *p* < 0.05 was considered statistically significant.

## 5. Conclusions

In conclusion, treatment with IL-10 and TGF-β significantly suppressed the induction of IL-1β and TNF-α, and this suppression was sustained. Thus, IL-10 and TGF-β may slow the progression of IDD by suppressing the release of inflammatory mediators from degenerative IVD cells, by promoting degradation and suppressing translation of TNF-α and IL-1β mRNA. Additionally, TGF-β may share some anti-inflammatory properties with IL-10. As IL-10 and TGF-β blocked the cascade of inflammatory cytokines produced by degenerative IVDs, this likely limited the development of additional inflammatory responses such as increased MMP production. These activities would then inhibit catabolic processes that cause extracellular matrix degradation while enhancing synthesis and maintenance of the extracellular matrix, preserving IVD cell functions, ultimately inhibiting the progression of IDD. Thus, IL-10 and TGF-β have potential biotherapuetic use for the treatment of IDD.
